# Detecting molecular subtypes from multi-omics datasets using SUMO

**DOI:** 10.1016/j.crmeth.2021.100152

**Published:** 2022-01-14

**Authors:** Karolina Sienkiewicz, Jinyu Chen, Ajay Chatrath, John T. Lawson, Nathan C. Sheffield, Louxin Zhang, Aakrosh Ratan

**Affiliations:** 1 Center for Public Health Genomics, University of Virginia, Charlottesville, VA 22908, USA; 2 Department of Mathematics and Computational Biology Program, National University of Singapore, Singapore 119076, Singapore; 3 Department of Biochemistry and Molecular Genetics, University of Virginia, Charlottesville, VA 22908, USA; 4 Department of Biomedical Engineering, University of Virginia, Charlottesville, VA 22908, USA; 5 Department of Public Health Sciences, University of Virginia, Charlottesville, VA 22908, USA; 6 University of Virginia Cancer Center, Charlottesville, VA 22908, USA; 7 These authors contributed equally; 8 Lead contact

## Abstract

We present a data integration framework that uses non-negative matrix factorization of patient-similarity networks to integrate continuous multi-omics datasets for molecular subtyping. It is demonstrated to have the capability to handle missing data without using imputation and to be consistently among the best in detecting subtypes with differential prognosis and enrichment of clinical associations in a large number of cancers. When applying the approach to data from individuals with lower-grade gliomas, we identify a subtype with a significantly worse prognosis. Tumors assigned to this subtype are hypomethylated genome wide with a gain of AP-1 occupancy in demethylated distal enhancers. The tumors are also enriched for somatic chromosome 7 (chr7) gain, chr10 loss, and other molecular events that have been suggested as diagnostic markers for “*IDH* wild type, with molecular features of glioblastoma” by the cIMPACT-NOW consortium but have yet to be included in the World Health Organization (WHO) guidelines.

## In brief

Sienkiewicz et al. present a computational method that performs joint factorization of multiple patient similarity networks to identify molecular subtypes of a disease. It can handle noisy and missing data effectively without imputation. The method consistently identifies subgroups with significantly different prognoses and enrichment of clinical associations on many datasets.

## Graphical abstract

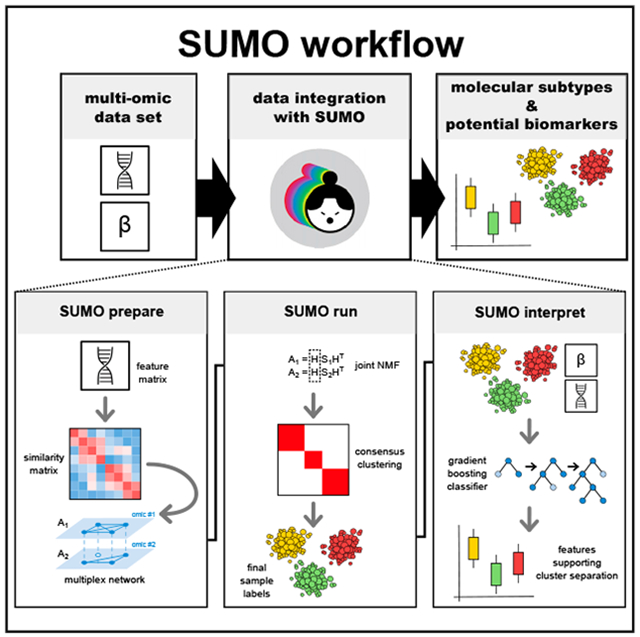

## INTRODUCTION

Biotechnologies for large-scale molecular studies of genetic diseases have advanced significantly. High-throughput assays are now available to measure RNA expression, DNA methylation, and metabolite concentration in tissues. Given that each assay reveals a snapshot of certain cellular aspects of a disease, integrative analyses are often necessary for a complete understanding of its molecular etiology and for discovering its molecular subtypes and biomarkers (Prasad et al., 2016).

Molecular typing through clustering has traditionally focused on gene expression. In studies with multiple data types, a popular strategy is to concatenate feature matrices from the various data types and then operate on the resulting matrix. This approach allows use of existing clustering techniques but requires cross-data type normalization and feature selection in individual data types before concatenation, possibly biasing the results. More sophisticated methods (e.g., iCluster [Shen et al., 2009], iClusterPlus [Mo et al., 2013], and Bayesian consensus clustering [Lock and Dunson, 2013]) model the probabilistic distribution of each data type and infer subtypes by maximization of the likelihood of the observed data. However, these methods require a feature selection step and make strong assumptions about the data.

More recent methods for clustering multi-omics data focus on distances between samples in lieu of clustering on the feature matrices. For example, PINS (Nguyen et al., 2017) clusters an average connectivity matrix based on the sample connectivity observed in the different data types. SNF (Wang et al., 2014) creates a fused network of individuals using a metric fusion technique and then partitions the data using spectral clustering. A more recent method, NEMO (Rappoport and Shamir, 2019b), calculates an average similarity matrix and then detects the clusters using spectral clustering. A comprehensive review of multi-omics and multi-view methods for subtyping diseases is presented by Rappoport and Shamir (2018).

The existing approaches have a few limitations. First, all approaches mentioned above, except for NEMO, require that data are available for every sample and every data type, which is unlikely for most biological studies. If data are incomplete, missing values can be imputed, but that is often computationally challenging for genome-wide analyses. Second, these methods often rely on randomization to overcome computational challenges. Although randomization can assist with finding a solution quickly, it has implications for the robustness of the method. Last, statistical methods have the advantage of being able to include biological knowledge as priors. However, they often assume a parametric normal or gamma distribution of the data to make the parameter estimation tractable. Such an assumption is often not realistic and again leads to poor performance, as demonstrated in a recent comprehensive assessment of the methods for drug response prediction (Chen and Zhang, 2021).

**Figure 1.**
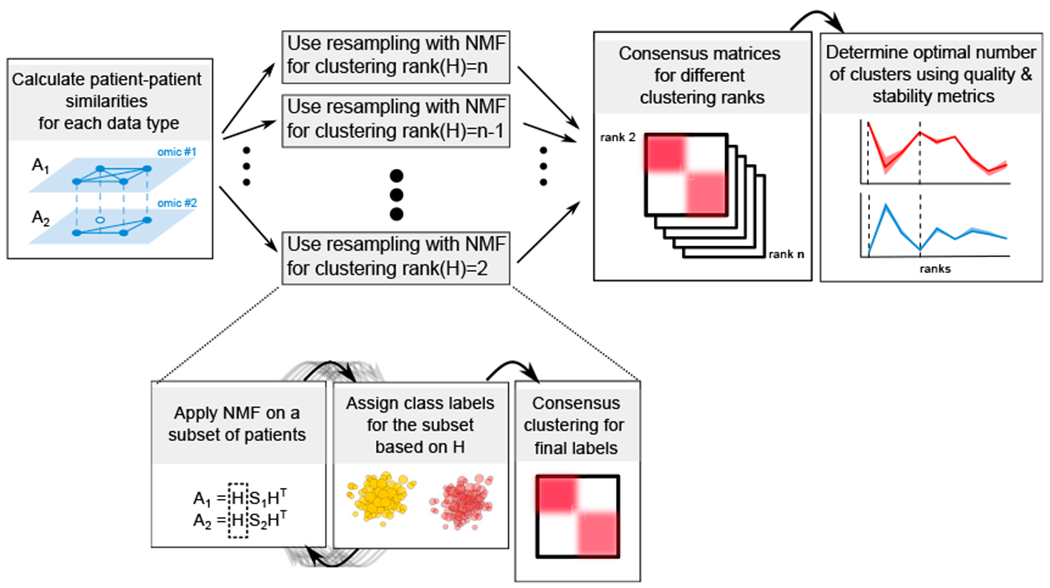
Summarized SUMO workflow SUMO uses NMF to determine consensus class labels for a varying number of clusters. Multiple quality and stability metrics are then used to determine the optimal clustering rank. We expand on how resampling with NMF is applied for 2 clusters. The gray arrows represent subsampling of the dataset.

Here, we present a data integration framework based on non-negative matrix factorization (NMF) and showcase an implementation called SUMO (https://github.com/ratan-lab/sumo) that can integrate continuous data from multiple data types to infer molecular subtypes. SUMO effectively handles missing data and produces robust clusters by using a resampling-based approach (Figure 1). Throughout the study, whenever appropriate, we compare SUMO v.0.2.6 with the iClusterBayes extension of iClusterPlus v.1.26, LRAcluster v.1.0 (Wu et al., 2015), MCCA from PMA package v.1.2.1 (Witten and Tibshirani, 2009), NEMO v.0.1 (Rappoport and Shamir, 2019b), PINSPlus v.2.0.5 (Nguyen et al., 2017), SNF v.2.3 (Wang et al., 2014), and CIMLR v.1.0 (Ramazzotti et al., 2018). We use a recent benchmark (Rappoport and Shamir, 2018) and datasets from TCGA and METABRIC (Curtis et al., 2012) to show that SUMO is consistently among the best methods in identifying groups of individuals with significantly differential prognosis and enrichment of clinical associations. Using simulation, we also compare SUMO with the other methods regarding the ability to cluster noisy datasets, respond to perturbations, and handle missing information.

We apply SUMO to multi-omics datasets from individuals diagnosed with lower-grade glioma (LGG). Diffuse low-grade and intermediate-grade gliomas together make up the LGGs (World Health Organization grades II and III), a diverse group of primary brain tumors with highly variable clinical behavior. Several studies have associated *IDH* somatic mutations with a more favorable course of the disease and have identified multiple subtypes with a poor clinical course (Eckel-Passow et al., 2015; Ceccarelli et al., 2016). We identify a single cluster of individuals with a significantly differential prognosis with SUMO. Individuals assigned to this cluster include all who were reported to have a poor clinical course in other studies and are enriched for genome-wide hypomethylation, somatic chromosome 7 (chr7) gain, and chr10 loss. Our findings support the recently proposed minimal clinical criteria for diagnosis of such diffuse astrocytic gliomas, which, despite their histological profiles, follow a more aggressive disease course (Brat et al., 2018). The remaining clusters recapitulate known subtypes in LGGs, highlighting the effectiveness of our approach.

## RESULTS

### SUMO improves performance with noisy and incomplete data

**Figure 2.**
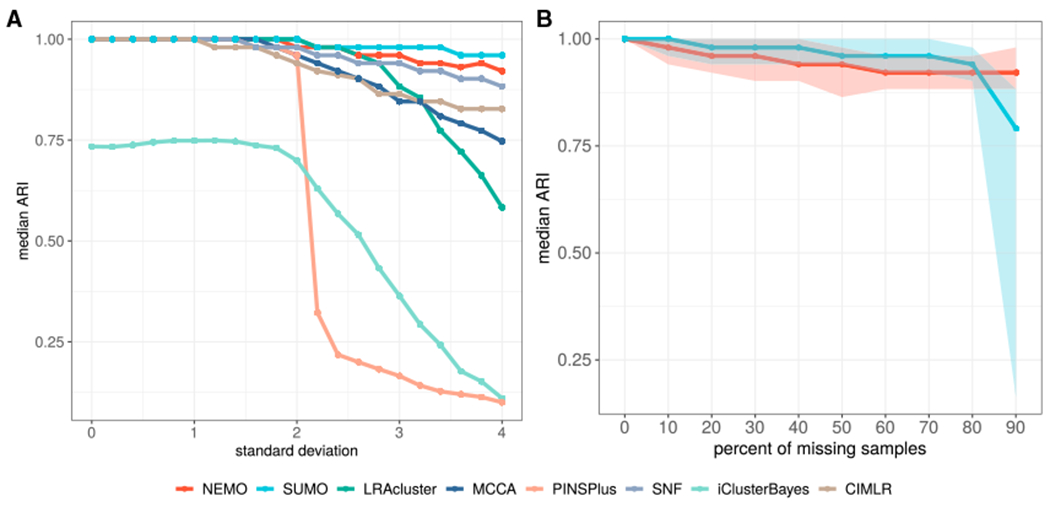
Accuracy of the eight methods on noisy data and missing values (A) All methods show reduced median accuracy with an increasing amount of noise, but SUMO exhibits better performance over a wide range. (B) SUMO shows a higher median ARI compared with NEMO for most data points when missing data are simulated in one of two data types. Error ribbons display the minimum and maximum ARI values.

We performed several simulations to compare the performance of the various methods on noisy datasets by varying the data noise level and the fraction of missing data that were generated by a procedure given in Figure 2. Figure S1A shows the experimental setup for the first simulation, where we increase the noise in one data type while keeping a moderate amount of noise in the other data type. The results in Figure 2A show that all methods exhibit a median decrease in accuracy with an increase in noise. SUMO has the highest median adjusted Rand index (ARI) and the least variance (Figure S1B) for all levels of noise.

Using the same setup, we compared SUMO with NEMO regarding their ability to accurately classify samples with missing data. Other methods do not handle missing data and so were not included in this comparison. In this experiment, we removed a random fraction of samples from one data type while preserving the data in the other data type. SUMO shows a higher median ARI compared with NEMO for most data points (Figure 2B).

### Performance of SUMO on a recent benchmark

We compared SUMO with several other methods using a recently published benchmark (Rappoport and Shamir, 2018). The benchmark consists of methylation, gene expression, and microRNA (miRNA) expression data from 10 cancer types sequenced as part of the TCGA project. As in the original benchmark, we evaluated each method for its ability to identify a subtype that shows significantly differential survival and is enriched for clinical annotations. We chose or calculated parameters for the methods, as suggested by the authors, without considering the survival and clinical parameters that are used for assessment. Data preprocessing for SUMO included applying a variance-stabilizing transformation (for count data) or converting beta values to M values (for methylation dataset), followed by feature standardization (as described in STAR Methods). For the remaining tools, we applied appropriate data preprocessing steps according to parameters specified by the authors of the benchmark.

**Figure 3.**
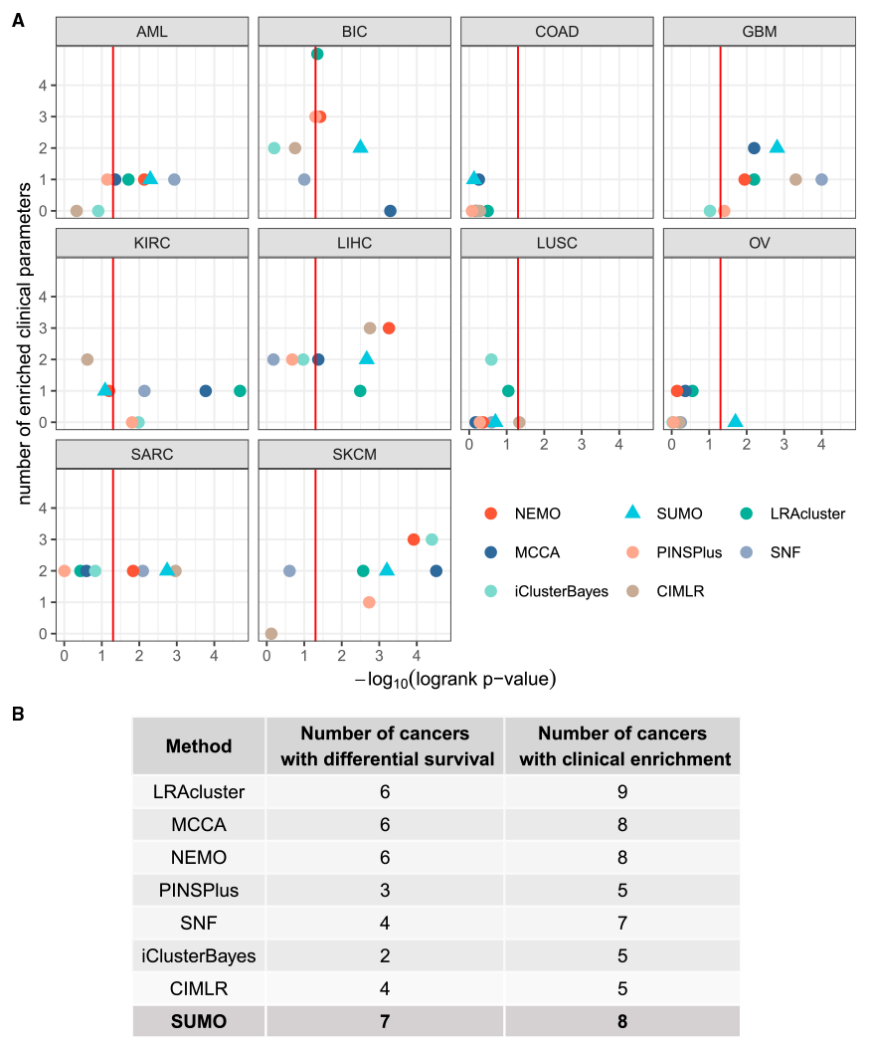
Benchmark results for the TCGA datasets (A) The vertical line indicates p = 0.05 for the log rank test, which is shown on the x axis. The y axis shows the number of clinical labels that were found to be enriched in at least one of the detected subtypes. SUMO results are shown using a triangle. (B) Summary of results from the benchmark analysis. We report the number of cancers for which at least one cluster had a significantly different prognosis (first column) that had at least one enriched clinical label (second column).

Figure 3A depicts the performance of the various methods on the data from the different cancer types. With respect to survival, SUMO had the total best prognostic value (sum of −*log*_10_ p = 18.88), with MCCA being the second best with 17.48. However, the sum of p values can be biased because of outliers, so we also counted the number of datasets for which a method’s solution obtains significantly different survival (p < 0.05) (Figure 3B). As with the original benchmark, we also evaluated whether at least one of the clusters was enriched for at least one of the clinical labels. The p values for the log rank test were calculated using permutation tests, enrichment for discrete parameters was calculated using the χ^2^ test for independence, and enrichment for numeric parameters was calculated using the Kruskal-Wallis test. The p values for clinical enrichment were corrected using Bonferroni correction. We also compared the p values of the log rank test when all data types were integrated to the p values when individual data types were considered for subtyping using spectral clustering. As we show in Figure S2A, integration of data types leads to an increase in the overall distribution of the Cox p values. Additionally, we used gene expression and PROGENy (Schubert et al., 2018) to calculate pathway activity scores for 14 signaling pathways. By applying the Kruskal-Wallis test, we confirmed that at least one pathway was differentially activated between clusters for each tool. More detailed information about datasets used in the benchmark, performance of specific tools, and activity of different pathways can be found in Tables S1A–S1C, respectively.

SUMO outperformed the other approaches in this benchmark, finding at least one cluster with significantly different survival in 7 of the 10 cancers analyzed. For colorectal cancer and lung squamous cell carcinoma, none of the methods identified a subtype that showed significant differential survival. SUMO is the only method to find a subgroup of individuals with ovarian cancer with a significant differential survival (Figure S2B). This group of individuals with a poor prognosis is enriched for those with mesenchymal tumors that are known to lead to worse outcomes (Cancer Genome Atlas Research Network, 2011). The scripts to reproduce the comparison of the various methods along with the instructions are available at https://github.com/ratan-lab/sumo_analysis.

All methods with the exception of iClusterBayes identified at least one cluster in glioblastoma (GBM) with a significantly differential prognosis. We used these GBM data to investigate the reproducibility and robustness of the methods; i.e., whether sample labels, the p values for the log rank test, or the number of enriched clinical parameters change if we changed the seed to the random number generator used by the methods and the assessment calculations. We ran each method 10 times using random seeds and found that the methods were stable to different extents on these data (Figure S2C). NEMO gave the same result in each of the 10 runs, whereas SUMO showed small deviations in the p values for survival, but the remaining methods showed variation in the p value of the log rank test and the χ^2^ test used to assess the enrichment of clinical parameters. PINSPlus results varied significantly in terms of survival and enrichment of clinical labels. It is likely that most of these methods, with the exception of NEMO, CIMLR, and iClusterBayes, would benefit from a resampling approach similar to that used by SUMO to generate more consistent subgroups in the data.

### SUMO applied to all TCGA cancer datasets

The benchmark from Rappoport and Shamir (2018) contains imputed and pre-filtered data from three different modalities: gene expression, DNA methylation, and miRNA expression for 10 cancers sequenced by TCGA. To further assess the performance of SUMO, we downloaded the harmonized gene expression and DNA methylation datasets for 34 cancers sequenced by TCGA from UCSC Xena, along with the miRNA expression for the 32 cancers where the data were available. We also downloaded two breast cancer datasets from METABRIC. We then used SUMO and the other seven methods to subtype each cancer based on the available modalities without any imputation. Detailed information about the datasets used in this analysis can be found in Tables S1D and S1E.

For each cancer, we again chose or calculated parameters for the methods, as suggested by the authors, without considering the survival parameters. We then used a log rank test to assess whether at least one of the subtypes showed significantly differential survival. A log rank test was performed using overall survival (OS) for all TCGA cancers except diffuse large B cell lymphoma (DLBC), testicular germ cell tumors (TCGT), thymoma (THYM), and pheochromocytoma and paraganglioma cancer (PCPG). Based on the recommendations by Liu et al., 2018, we used progression-free survival for DLBC, TCGT, and THYM. A survival analysis was not run for PCPG because none of the clinical endpoints are recommended for such an analysis. Disease-free survival data were used for METABRIC datasets. The p values for the log rank test were calculated using permutation tests.

**Figure 4.**
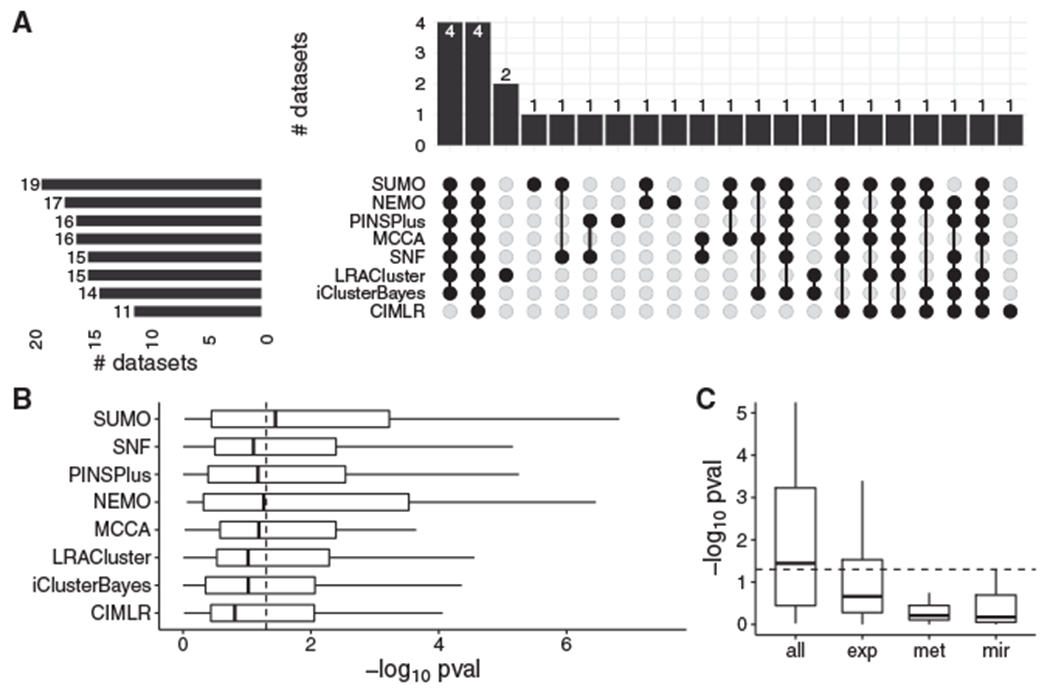
Comparison of the methods used on the TCGA and METABRIC datasets (A) Summary of results from the analysis in an UpSet plot. We report the number of cancers for which at least one cluster had a significantly different prognosis in the left panel. The number of datasets with overlap between the compared methods is shown in the top panel. (B) The vertical line indicates p = 0.05 for the log rank test, which is shown on the x axis using a −*log*_10_ scale. We plot the −*log*_10_ of the p values of the log rank test for all methods. (C) We compare the p values of the log rank test for each data type (exp, gene expression; met, DNA methylation; mir, miRNA) with the p values when all data types are integrated.

SUMO finds at least one significantly different prognostic cluster (p < 0.05) in 19 cancers, which is the most among the compared methods, followed by NEMO, which finds prognostic clusters in 17 cancers (Figure 4A). All methods identify at least one differentially prognostic cluster for four datasets (ACC, LGG, METABRIC Discovery, and METABRIC Validation), whereas such differentially prognostic clusters are identified by a single method for six datasets (Figure 4A). Overall, SUMO had the total best prognostic value (sum of −*log*_10_ p = 89.8) and the best median prognostic value (−*log*_10_ p = 1.44) among the methods compared in this analysis (Figure 4B). For nine of the datasets, SUMO has the lowest p values for the log rank test, followed by LRACluster, which has the lowest p values for seven datasets. For SUMO, the integration of data types improved its ability to identify differentially prognostic clusters, as determined by the increase in the median of Cox −*log*_10_ p values (Figure 4C) when the various data types are integrated.

We also used the gene expression data for each of the 34 cancers with PROGENy to calculate the pathway activity scores for 11 signaling pathways. We report the p value of the Kruskal-Wallis test to determine whether the pathway is differentially activated in at least one of the subtypes determined by SUMO in Table S1E. For all cancers, at least one of the pathways is differentially activated in at least one of the subtypes determined by SUMO, highlighting that SUMO identifies biologically relevant subtypes. Besides the selected number of subtypes, the negative logarithm of the p value for the log rank test, and the p value of the Kruskal-Wallis test, we report the average silhouette score based on the consensus matrix in Table S1D. The mean silhouette score for 25 of the 34 cancers is higher than 0.9, showing that the class labels generated using the resampling strategy are robust for several datasets.

### SUMO analysis of TCGA-LGG identifies a cluster of individuals with poor prognosis

The 2016 World Health Organization (WHO) classification of diffuse gliomas recommends use of *IDH* mutation status to separate *IDH* mutant astrocytoma and oligodendroglioma from *IDH* wild type astrocytoma, which has a worse prognosis (Louis et al., 2016). Several integrative approaches have been applied since then to further understand the molecular heterogeneity and subtypes in gliomas. The largest study of diffuse grade II-III-IV gliomas to date used TumorMap (Newton et al., 2017) to integrate gene expression and DNA methylation data from around 1,000 individuals, and they, too, identified *IDH* status as the primary driver of two macro-clusters (Ceccarelli et al., 2016). The authors concluded that the *IDH* mutant gliomas were further composed of three coherent subgroups: (1) the Codel group, consisting of LGGs with 1p/19q codeletion; (2) the G-CIMP-low group, including gliomas without 1p/19q codeletion with relatively low genome-wide DNA methylation; and (3) the G-CIMP-high group, including gliomas without 1p/19q codeletion with higher global levels of DNA methylation. They also concluded that the *IDH* wild type gliomas segregated into three subgroups: (1) classic-like, exhibiting a classical gene expression signature; (2) mesenchymal-like, enriched for mesenchymal subtype tumors; and (3) pilocytic astrocytoma (PA)-like, enriched for tumors with molecular similarity to grade I PA.

**Figure 5.**
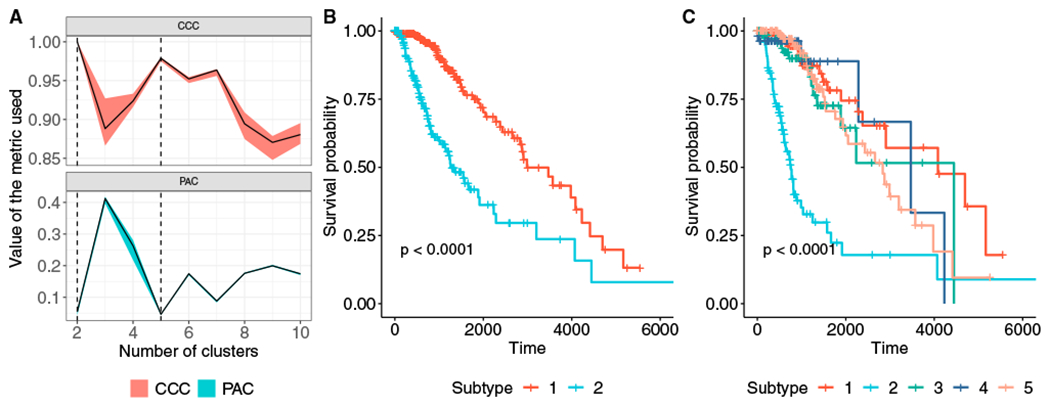
SUMO detects a single cluster showing differential prognosis in TCGA LGG (A) The two metrics used to decide the optimal number of clusters for the LGG dataset. We use the proportion of ambiguously clustered pairs (PAC) (lower is better) and the cophenetic correlation coefficient (CCC) (higher is better) to select 2 and 5 as the optimal numbers of clusters, shown using dashed lines. (B and C) KM analysis of the subtypes detected by SUMO when 2 and 5 clusters are selected, respectively. We report the p values of the log rank test.

The cIMPACT-NOW (the Consortium to Inform Molecular and Practical Approaches to CNS Tumor Taxonomy) initiative was established to evaluate and recommend changes to future CNS tumor classifications based on consensus review of novel diagnostically relevant data (Louis et al., 2017). In seven separate updates since its inception, the initiative has issued interim recommendations for CNS tumor classifications. We decided to apply SUMO to subtype the LGGs as a case study with the intent to evaluate the robustness and relevance of known and proposed glioma subtypes. We used SUMO to integrate the processed level 3 gene expression, DNA methylation, and miRNA expression data for the TCGA-LGG cohort downloaded from the UCSC Xena platform (Goldman et al., 2019) (and preprocessed as described in the Data preprocessing part of the STAR Methods). We evaluated the solutions with 2–19 clusters according to the proportion of ambiguously clustered pairs (PACs) (Şenbabaoğlu et al., 2014) and the cophenetic correlation (Hutchins et al., 2008; see STAR Methods for details). The PAC values suggest that the individuals can be partitioned into 2 or 5 clusters, with both solutions being stable (Figure 5A).

Figure 5B shows the Kaplan-Meier survival analysis for the 2 clusters identified by SUMO. The cluster of individuals who show a better prognosis include a majority of *IDH* mutant LGGs with 1p/19q codeletion and the majority of the *IDH* mutant LGG without 1p/19q codeletion with higher global levels of DNA methylation. Figure S3A summarizes the association of the 2 clusters with mutations in known driver genes, existing supervised classifications, and histological parameters. We focus on the solution with 5 clusters for the remainder of this study (see Table S1F for final classification labels).

Figure S3B shows a heatmap of the similarity matrices and Sankey plots comparing the clustering performed on each data type with the clustering done after integration of the three data types. We annotate the clusters determined using spectral clustering as annotations on the top of the heatmap, whereas the clusters determined by SUMO based on integrative clustering are shown on the right. These plots illustrate how the data integration incorporates the information from each of the data types and generates more fine-grained structures. As an example, the individuals in group 2 based on methylation alone are partitioned into two separate subtypes when expression and methylation are considered together.

**Figure 6.**
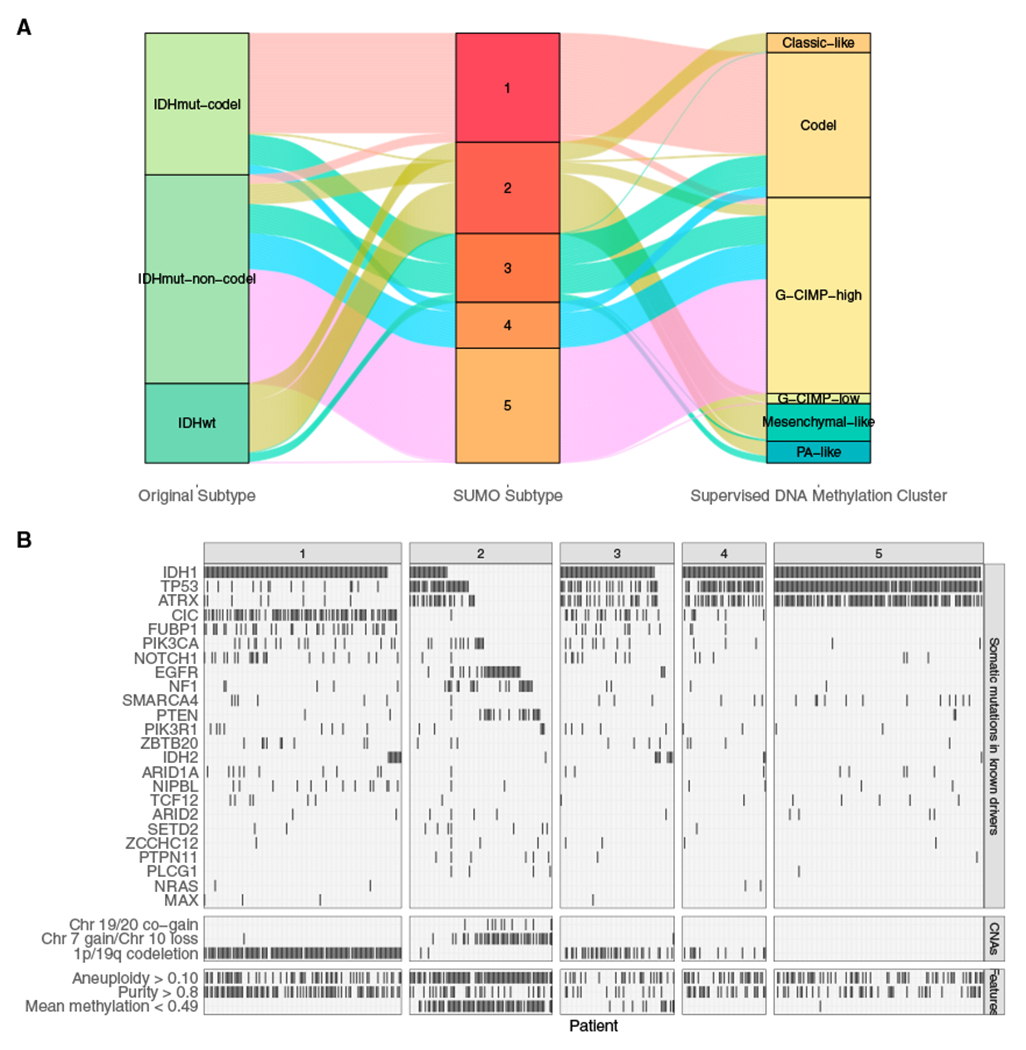
Features of tumors assigned to the various subtypes (A) Sankey plot comparing the assignment by SUMO to the subtypes assigned using the WHO recommendation and the supervised DNA ,ethylation clusters assigned by Ceccarelli et al., 2016. (B) Oncoplot showing enrichment of mutations, copy number aberrations, and molecular features of LGGs stratified by SUMO subtypes.

Figure 5C shows the Kaplan-Meier survival analysis for the 5 clusters as identified by SUMO. Individuals assigned to subtype 2 show a significant differential prognosis with a median survival of 758 days. 76 of 80 samples in subtype 2 were labeled as classic-like, mesenchymal-like, and C-GIMP low and reported to have a poor clinical course by Ceccarelli et al., 2016) (Figure 6A). We find that subtype 2 is enriched for individuals who are *IDH* wild type and who were significantly older at the age of diagnosis (Tukey HSD test, p < 0.05 for all pairwise comparisons). Subtype 2 is also enriched for grade III tumors (odds ratio [OR] 6.28; 95% confidence interval [CI], 3.40–11.59) and significantly enriched for anaplastic astrocytomas (p < 10^−5^); it is also enriched for samples with a high percentage of aneuploidy (Tukey HSD test, p < 0.05 for all pairwise comparisons).

Figure 6B summarizes these associations in an oncoplot. Subtype 2 is enriched for point mutations and amplifications of the epidermal growth factor receptor (*EGFR*) oncogene on chr7. Somatic aberrations in the *EGFR*, including amplification and activating point mutations, occur in ~57% of grade IV gliomas but are relatively uncommon in LGGs (Brennan et al., 2013). However, 55 of the 109 individuals assigned to subtype 2 show chr7 gain (and, hence, amplification of the *EGFR*) and chr10 loss, which leads to deletion of the *PTEN* gene, a known tumor suppressor. According to the WHO guidelines from 2016, chr7 gain and/or chr10 loss are not considered in the diagnosis of grade II/III gliomas, although other studies have suggested that these events are clinically relevant. Recent recommendations from the cIMPACT-NOW consortium suggest that *EGFR* amplification and combined chr7 gain and chr10 loss as well as *TERT* promoter mutation can be used to diagnose *IDH* wild type (WT) grade II/III gliomas that are likely to follow a more aggressive clinical disease course (Brat et al., 2018). Our results support the proposed inclusion of additional diagnosis criteria, in particular chr7 gain and chr10 loss, which could lead to reclassification of several LGGs into GBMs (Stichel et al., 2018).

We used ELMER (Silva et al., 2019) in an unsupervised mode to compare subtype 2 tumors with the other LGGs. ELMER identified 382 probes overlapping putative distal enhancers that were hypomethylated in subtype 2 samples (adjusted p < 0.01; methylation difference between means of the groups, >0.3). The motifs with the highest enrichment around the 382 putative distal enhancers correspond to the Fos and Jun transcription factor gene families. Fos genes encode leucine zipper proteins that can dimerize with proteins of the JUN family, forming the early response transcription factor complex AP-1. Therefore, FOS proteins have been implicated as regulators of cell proliferation, differentiation, and transformation (Mehta and Lo Cascio, 2018). More specifically, we find that the expression of *FOSL1*, which contributes to regulation of placenta development, is significantly higher in subtype 2 tumors, and higher expression of the gene is associated with a worse prognosis (Kubota et al., 2015). These results are in agreement with other published studies that show that AP-1 binds to demethylated regions in G-CIMP-low tumors, but we find this to be true for all samples assigned to subtype 2 (Souza et al., 2018).

**Figure 7.**
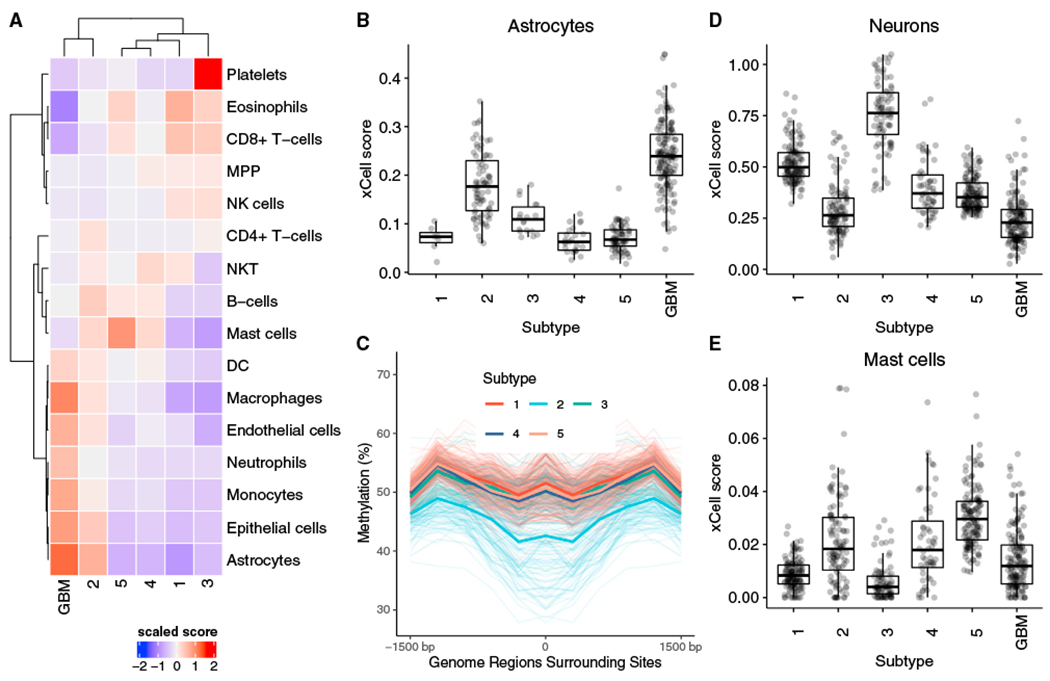
Subtype 2 shows similarities to GBM, and subtype 3 is enriched for neurons and subtype 5 for mast cells. (A) Heatmap showing the mean xCell enrichment scores for the LGG subtypes and GBM corresponding to parental cell types expected in the brain, with subtype 2 and GBM sharing enrichment of several cellular populations. (B) Astrocytomas assigned to subtype 2 show higher xCell scores compared with astrocytomas that are assigned to the other LGG subtypes. (C) Tumors assigned to subtype 2 show lower methylation and higher regulatory activity at astrocyte-specific elements. The mean methylation levels are shown using a dark line. (D) Tumors in subtype 3 are enriched for neuronal cells. (E) Tumors in subtype 5 are enriched for mast cells.

Because tumors are a complex milieu of numerous cell types, we hypothesized that the microenvironment plays an important role in determination of these subtypes. To investigate this, we downloaded the xCell scores corresponding to the enrichment of 64 different immune and stromal cell types in these TCGA samples (Aran et al., 2017). Hierarchical clustering of the mean enrichment scores for the various cell types that are expected to be present in the brain (Figure 7A) shows that the cellular profile of subtype 2 tumors is more similar to GBMs than to the other LGGs. More importantly, astrocytomas assigned to subtype 2 have higher enrichment scores for astrocytes, similar to those calculated for GBM samples and significantly higher than astrocytomas assigned to the other subtypes (Figure 7B). xCell scores are derived from gene expression, but we observe similar results in analysis of methylation data using MIRA (Lawson et al., 2018). Subtype 2 samples show lower methylation and higher regulatory activity at astrocyte-specific elements (Figure 7C) compared with the other subtypes.

Among other subtypes, subtype 1 is enriched for oligodendrogliomas (p < 1.0 × 10^~5^), mutations in the *TERT* promoter and high expression of *TERT* (Tukey HSD test, p < 0.05 for all pairwise comparisons), high tumor purity (Tukey HSD test, p < 0.05 for all pairwise comparisons), 1p/9q codeletion, and mutations in *CIC*, a known tumor suppressor. 128 of the 130 individuals in subtype 1 have a methylated promoter for *MGMT* (post hoc test of residuals for χ^2^ test, p < 1.0 × 10^−5^). *MGMT* promoter methylation is associated with a better response to alkylating chemotherapy, suggesting that individuals assigned to subtype 1 are more likely to respond to temozolomide (Rivera et al., 2010).

Subtype 3 is enriched for the neural (NE) subtype detected in previous gene expression studies. The NE subtype has been related previously to the tumor margin, where increased normal NE tissue is likely to be detected (Gill et al., 2014). Consistent with this hypothesis, we find that the tumors assigned to subtype 3 have lower tumor purity (Tukey HSD test, p < 0.05 for all pairwise comparisons except with subtype 5) and a high enrichment score for neurons (Figure 7D). Subtypes 4 and 5 are enriched for G-CIMP-high samples, although subtype 5 is enriched for mutations in *ATRX* (post hoc test of residuals for χ^2^ test, p < 10^−5^) and shows a higher enrichment for mast cells (Figure 7), which are known to induce release of selective inflammatory cytokines, such as interleukin-4 (IL-4), with anti-glioma activity, leading to an improved prognosis (Benedetti et al., 2000).

## DISCUSSION

We present an approach to integrate multi-omics datasets using the NMF of patient-similarity networks. Similar to other similarity-based methods, such as SNF, we first transform information from each data type into a separate patient-similarity network, which allows us to preserve and handle data-type-specific properties. We then use a joint factorization to calculate a shared representation of the samples in a lower-dimension subspace. Our implementation, SUMO, additionally enforces sparsity on this representation, making it well suited for unsupervised learning. Furthermore, we use a resampling technique in conjunction with consensus clustering to detect the optimal number of clusters and assess the stability of the generated clusters. Our validation experiments show that this resampling strategy has the potential to improve the output from other subtyping methods as well.

The importance of integrative clustering has been recognized for several years, and several methods have been developed to exploit the increasing number of multidimensional datasets. SUMO improves existing methods in its ability to handle noisy and missing data. We compared SUMO with several existing methods for integrative clustering. SUMO produces consistently reproducible results on a recently published benchmark. The benchmark uses differential survival and enrichment of a small number of clinical labels in the resulting clusters as metrics for assessment of subtyping methods. However, subtypes of a disease that are biologically different can lead to similar survival. For example, SUMO does not detect a subtype with a significantly different prognosis in colorectal adenocarcinoma, but the determined subtypes show significant differences in the activity of the various signaling pathways. Furthermore, using survival as a metric is biased to known cancer subtypes, which may have been used in treatment decisions. SUMO has the total best prognostic value compared with the other methods on this benchmark and the TCGA datasets, but evaluating multi-omics clustering methods remains a challenge. Using real datasets and established benchmarks is a reasonable approach to such comparisons, although it is important to remember that they are limited as well. In this study, we report the p values of the log rank test using exact permutation tests because p values based on the χ^2^ approximation are highly inaccurate in evaluating clustering solutions on real cancer datasets (Rappoport and Shamir, 2019a).

When applying SUMO to subtype LGGs, we identified a single subtype with a differential prognosis compared with the other subtypes. We show that this subtype includes all previously studied groups of individuals with features that are associated with a poor outcome. Like GBM, gain of chr7, loss of chr10, and global hypomethylation appear to be hallmarks of this subtype. Our analyses suggest that LGGs assigned to subtype 2 should be treated more aggressively and potentially reclassified as GBM. These findings are in agreement with the recommendations from the cIMPACT-NOW consortium, which suggests that *EGFR* amplification and combined chr7 gain and chr10 loss as well as *TERT* promoter mutation can be used to diagnose the *IDH* WT grade II/III gliomas that are likely to follow a more aggressive clinical disease course.

The choice of samples and data types can have a significant effect on the inferred subtypes, making determination of subtypes challenging. For example, a large study of diffuse grade II-III-IV gliomas by Ceccarelli et al. (2016) classified a subset of *IDH* WT LGG samples as “PA-like” based on the molecular similarity to grade I PA and improved prognosis compared with the other *IDH* WT samples. However, they also reported that several GBM (grade IV) samples with a poor prognosis were assigned to the same cluster as the PA-like samples based on the CpG methylation markers, highlighting that tumor grade provided prognostic value independent of subtype and age. We found that PA-like samples from Ceccarelli et al. (2016) are classified by SUMO primarily into two different groups based on gene expression and miRNA expression. The assignment correlates with the tumor grade, with most grade III tumors being assigned to subtype 2, which is characterized by a poor clinical course. This example highlights the need for integration of clinical observations and outcomes with molecular information in determination of clinical subtypes. Even though we use our approach to cluster samples, additional constraints can be used to adapt the approach for semi-supervised applications, as suggested in other studies using NMF (Choo et al., 2015).

SUMO is available as a Python package, which includes modules to construct patient-similarity networks and infer molecular subtypes. A common post hoc analysis of molecular subtyping is identification of features that can be used as markers or surrogates for the various subtypes. SUMO includes a mode to build a tree-based model that can predict the importance of each feature for each of the detected subtypes. For example, we identified a clinically relevant subtype of LGG with a differential prognosis compared with the other subtypes. According to our analysis, the non-CpG island methylation probes in proximity to the gene *CLCF1* are the best markers for the subtype. Figure S3C shows the beta values of the samples for the three methylation probes that have the highest explanatory values for the classifier.

### Limitations of the study

In this study, we compare SUMO with several methods that integrate continuous genomic data types to discover biologically relevant molecular subtypes. The metrics used in these assessments have their limitations and can reflect adversely on the performance of methods with additional objectives beyond determination of subtypes. Even though we can integrate categorical and ordinal data types using SUMO, we did not benchmark SUMO against the other methods for those data types. The current implementation of SUMO has a few limitations. For example, it can be slower than competing methods for large datasets because it uses consensus clustering of multiple NMF decompositions to assign the final labels. Even though the current implementation can train a gradient boosting classifier to identify features that can act as biomarkers for the assigned clusters, the implementation requires large amounts of memory.

## STAR★METHODS

### RESOURCE AVAILABILITY

#### Lead contact

Further information and requests for resources should be directed to and will be fulfilled by the lead contact, Aakrosh Ratan (ratan@virginia.edu).

#### Materials availability

This study did not generate new unique reagents.

#### Data and code availability

- This paper analyzes existing, publicly available datasets processed and hosted on UCSC Xena at https://xenabrowser.net/datapages/?hub=https://tcga.xenahubs.net:443. Information also listed in the key resources table.
- SUMO is implemented in python and freely available in the form of a command-line tool on GitHub (https://github.com/ratan-lab/sumo) and at The Python Package Index (https://pypi.org/project/python-sumo). The official documentation including a tutorial for SUMO is available at https://python-sumo.readthedocs.io. We used SUMO v0.2.6 in this study which is available from https://doi.org/10.5281/zenodo.5762331. The scripts to reproduce the comparison of the various methods along with the instructions are available at https://doi.org/10.5281/zenodo.5762339. Information also listed in the key resources table.
- Any additional information required to reanalyze the data reported in this paper is available from the lead contact upon request.

### METHOD DETAILS

We performed several simulations to compare the performance of the various methods on noisy datasets with varying the data noise level and the fraction of missing data.

#### Simulated noisy dataset

Figure S1A shows the experimental setup for a simulation where we increase the noise in one data type while keeping a moderate amount of noise in the other data type. We first generated a ‘ground truth’ feature matrix consisting of 200 samples and 400 features, with two distinctly separable clusters (isotropic Gaussian ‘blobs’ with a standard deviation of 0.5). Next, we simulated one data type by adding random noise from a Gaussian N(μ=0,σ=1.5) distribution to those clusters. We simulated another data type by adding noise to the clusters from a Gaussian distribution (N(μ=0) where the standard deviation is varied *σ* ⎞ (0, 4)). We then calculated the median ARI of the classification at each data point for 100 repetitions for each method compared in the experiment. The scripts to produce the simulated dataset and compare the various methods are available at https://github.com/ratan-lab/sumo_analysis.

#### Simulated missing dataset

To simulate missing information, we removed a random fraction of samples from one of the two data types selected at random while keeping corresponding sample data in the other data type. We again calculated the median ARI of classification at each data point for 100 repetitions for each method compared in the experiment. The scripts to produce the missing dataset and compare the various methods are available at https://github.com/ratan-lab/sumo_analysis.

#### Benchmark

We compared SUMO to several other methods using a recently published benchmark (Rappoport and Shamir, 2018). The scripts to compare the various methods on this benchmark is available at https://github.com/ratan-lab/sumo_analysis.

#### Method overview

The NMF technique aims to explain the observed data using a small number of basis components by factoring the data into the product of two non-negative matrices; one representing the basis components, and the other containing mixture coefficients (Paatero and Tapper, 1994). NMF has been successfully used as a clustering method in image and pattern recognition (Leuschner et al., 2019), text-mining (Chen et al., 2015), and bioinformatics (Wang et al., 2015). In this work, we used a variant of NMF called Symmetric NMF, in which the decomposition is done on a symmetrical matrix that contains pairwise similarity values between the data points instead of being done directly on the data points (Kuang et al., 2012). Symmetric NMF improves clustering quality compared to the traditional formulation.

Similar to NEMO and SNF, we preprocess, transform, and standardize the data before calculating the similarity between the samples for each data type separately. If all data types are measured for all *n* samples, the similarity between samples based on the *i*^*th*^ data type forms a *n* × *n* symmetric matrix *A_i_*. After that, we tri-factorize *A*_*i*_ ≈ *HS*_*i*_*H*^*T*^, where *H* is a non-negative *n* × *r* matrix, *S_i_* is a *r* × *r* non-negative matrix, and *r* (≪*n*) is the desired number of clusters. *H* in this decomposition is shared among the various data types and is a representation of the *n* samples in a *r*-dimensional subspace accounting for the adjacencies observed in all data types. Each row of *H* represents a sample, whereas each column of *H* denotes a cluster. We include an additional constraint to enforce sparsity of *H* in the factorization.

Lastly, we use multiplicative updates to solve the above factorization. Since the solution can be sensitive to the initial conditions and the input data, we run the solver multiple times on several subsets of samples using different initial conditions and use consensus clustering to assign the final labels and infer the optimal number of clusters (Figure 1). We describe these steps in detail below.

#### Data preprocessing

Data preprocessing involves (a) filtration, (b) transformation, and (c) normalization of each data type separately. The filtering process removes features that are not informative; for example, we remove genes with zero counts in most samples. Although our approach can handle missing values, removing features and samples with a large fraction of missing values (>10%) often speeds up computation and is recommended unless it removes a significant fraction of samples.

The transformation process is data-dependent. For instance, we use a variance-stabilizing transform to convert abundance in count data (as in RNA-seq) to yield a matrix of values that are approximately homoscedastic (with constant variance in the range of mean values). This transformation has the additional advantage of reducing the effect of outliers in the data. In the case of methylation data, we use of log2 ratio of the methylated to unmethylated count, also referred to as M-values (Du et al., 2010). If batch information is known, we use ComBat (Johnson et al., 2007) to adjust for batch effects in this step.

In the normalization step, we perform feature standardization to make the value of each feature in the data be zero-mean and unit variance. Our data-preprocessing is similar to other similarity-based methods such as SNF, but we do not require imputation to fill in missing data, which can be computationally intensive for genome-wide datasets.

#### The construction of similarity networks and matrices

Let *n* be the number of patient samples *s* that are found in the dataset of every data type and let *t* be the number of data types (e.g., gene expression or DNA methylation). In this step, we construct a similarity network *N*, represented by a set of *n*×*n* similarity matrices {*A*_1_,*A*_2_,⋯,*A*_*t*_}, where *A*_*k*_(*i,j*) = (*a*_*ij*_(*k*)) and *k* is used as an index for the data type. *a_ij_*(*k*) represents the similarity between two samples *s*_*i*_ and *s_j_* calculated from the features of the *k*^*th*^ data type, *k* = 1,⋯, *t*.

For each data type *k*, we assume its data is represented in a matrix (*f_ij_*) containing *n* sample rows and *p* feature columns. We calculate *A*_*k*_ as a radial basis function of the Euclidean distance $\rho (i,j)=\sum_{m=1}^{p}{\left(f_{im}-f_{jm}\right)}^{2}$ between the samples *x*_*i*_ and *x_j_*:

$$A(i,j)=\exp\left(-\frac{\rho^{2}(i,j)}{\mu \varepsilon_{i}\varepsilon_{j}}\right)$$

where *μ* is a hyperparameter and ε_*i*_ represents the average distance between *x*_*i*_ and its *K* nearest neighbors *N*_*K*_(*i*):

$$\varepsilon_{i}=\frac{\sum_{j\in N_{K}(i)}\rho (i,j)}{K}.$$

We set *μ* equal to 0.5 based on performance on simulated datasets and set the number of nearest neighbors *K* equal to 10% of the samples in the data type. The selection of *K* and *μ* change the emphasis on the local and global structure in the similarity graph and can affect the results. In Figures S4A and S5A, we apply the similarity kernel to a single feature dataset generated from a normal distribution with a mean of zero and a standard deviation of three. We can see that lower values of *μ* and higher values of *K* both increase the number of pairs with lower values in the similarity matrix. Varying these parameters does not have a significant effect when SUMO is applied to well-separated simulated datasets with a known number of clusters, even with large amounts of missing data or noise (Figures S4B and S5B). However, with real data, varying *μ* or *K* can change the optimal number of clusters as suggested by the two metrics PAC and CCC. Lower or higher extreme values of *μ* lead to a higher number of clusters being selected as optimal, as we show in Figure S4C. Similarly, the selection of K can also influence the optimal number of clusters in real data (Figure S5C), with lower values emphasizing the local neighborhoods of samples. We recommend setting *K* to $\frac{\#\mathrm{samples}}{\#\mathrm{clusters}}$ if the number of clusters is known.

The Euclidean distance is appropriate for normalized count datasets, such as for gene expression and DNA methylation data. However, depending on the data type and the application, different distances or similarity metrics may better represent sample relationships. For example, cosine similarity has been shown to be a better metric for the calculation of similarity between single cells in the single-cell sequencing for transposase accessible chromatin (scATAC-seq) (Cai et al., 2018). Currently, the SUMO package (see implementation details section) implements four alternative methods to create similarity matrices: Euclidean distance, cosine similarity, Pearson and Spearman correlation. All distance measures are subject to the following constraints: *a*_*ij*_(*k*) ∈ [0, 1] and *a*_*ii*_(*k*) = 1.

#### Joint tri-factorization of the similarity matrices

Each matrix *A*_*i*_ of the multiplex network *N* is symmetric and non-negative. We tri-factorize *A*_1_,*A*_2_, ⋯,*A*_*t*_ as follows:

$$A_{i}\approx HS_{i}H^{T},\;i=1,\cdots ,t,$$

in which *H* is a *n* × *r* matrix shared across the data types and *r* is the desired number of clusters such that *r* ≪ *n* (Figure S6B).

We compute the above tri-factorization by minimizing the following objective function:

(Equation 1) $$L=\sum_{i=1}^{t}\lambda_{i}{∥W_{i}\circ \left(A_{i}-HS_{i}H^{T}\right)∥}_{F}^{2}+\eta {∥H∥}_{F}^{2}$$

where ∘ denotes entry-wise multiplication for matrices, and *H* and *S*_*i*_ are both constrained to be non-negative. The first term of the objective function measures the divergences between *A*_*i*_ and *HS_i_H*^*T*^ using the Frobenius norm in each data type. For each data type, measurements may be not available for all the *n* samples, thus leading to missing entries in the matrix *A_i_*. We use *W_i_* to remove the missing values, where

$$W_{i}(x,y)=\{\begin{array}{cc} 1 & \mathrm{if}\;A_{i}(x,y)\;\mathrm{is available}\\ 0 & \mathrm{otherwise} \end{array}$$

Then we add another factor $\lambda_{i}=n_{i}^{-2}$ to account for the imbalance in the number of entries among *A*_*i*_(*i* = 1,…, *t*), where *n*_*i*_ is the number of samples for the *i*^th^ data type.

The second term of the objective function is used to enforce sparsity on the matrix *H*, and the hyperparameter *η* is used to balance the contribution of these two terms (see implementation details section for more information on *η* selection).

Note that the cost function in Equation 1 is convex in either but not both *H* and *S_i_*. The following multiplicative updates are used to solve the optimization problem given in Equation 1 (See the next section for details on the derivation of the rules).


$$S_{i}\leftarrow S_{i}\circ \frac{H^{T}\left(W_{i}\circ {\text{A}}_{i}\right)H}{H^{T}\left(W_{i}\circ \left(HS_{i}H^{T}\right)\right)H}$$

$$H\leftarrow H\circ \frac{\underset{i}{\sum^{\text{​}}}\lambda_{i}\left(W_{i}\circ A_{i}\right)HS_{i}}{\underset{i}{\sum^{\text{​}}}\lambda_{i}\left(W_{i}\circ \left(HS_{i}H^{T}\right)\right)HS_{i}+0.5\eta H}$$

As the algorithm iterates using the updates, *H* and *S_i_* converge to a local minimum of the cost function. We apply the above rules iteratively while alternating fixed matrices, keeping track of objective function value $L^{\left(i\right)}$ until it satisfies

$$\frac{\left|L^{\left(i+1\right)}-L^{\left(i\right)}\right|}{L^{\left(i+1\right)}}<\varepsilon$$

where ε is a predefined threshold, or the maximum number of allowed iterations is reached.

Since the solution is relatively sparse, we can assign each sample (represented by a row in *H*) to the cluster corresponding to the column that contains the maximum value, as depicted in Figure S6C. In practice, the solution can be sensitive to the input dataset and the initial conditions. We discuss the details of this in the Implementation details, but briefly, we run the above solver multiple times on subsets of the dataset and then use consensus clustering to get the final assignments.

#### Derivation of the multiplicative-update rules

For the objective function Equation 1, when we update matrix *S_i_*, matrices *H* and *S_j_*(*j* ≠ *i*) should be fixed, thus it would be an optimization problem about the matrix *S_i_*, that is,

$$\min{∥W_{i}\circ \left(A_{i}-HS_{i}H^{T}\right)∥}_{F}^{2},\;\mathrm{subject to}\;S_{i}\geq 0.$$

The corresponding Lagrange function is

$$L\left(S_{i}\right)=tr\left({\left(W_{i}\circ \left(A_{i}-HS_{i}H^{T}\right)\right)}^{T}\left(W_{i}\circ \left(A_{i}-HS_{i}H^{T}\right)\right)\right)-tr\left(B_{i}^{T}S_{i}\right),$$

where *B_i_* ≥ 0 is the Lagrange multiplier for *S_i_*, and *tr*(*X*) represent the trace of matrix *X*. Then

$$\frac{\partial L\left(S_{i}\right)}{\partial S_{i}}=-2H^{T}\left(W_{i}\circ \left(A_{i}-HS_{i}H^{T}\right)\right)H-B_{i}.$$

Let $\frac{\partial L\left(S_{i}\right)}{\partial S_{i}}=0$, thus

$$H^{T}\left(W_{i}\circ \left(HS_{i}H^{T}\right)\right)H-H^{T}\left(W_{i}\circ A_{i}\right)H=\frac{1}{2}B_{i},$$

and

$${\left(S_{i}\right)}_{jk}∙{\left(B_{i}\right)}_{jk}=0,$$

thus *S_i_* satisfies

$${\left(H^{T}\left(W_{i}\circ \left(HS_{i}H^{T}\right)\right)H-H^{T}\left(W_{i}\circ A_{i}\right)H\right)}_{j}∙{\left(S_{i}\right)}_{jk}=0.$$

We obtain the update formula for *S_i_* as follows:

$$S_{i}\leftarrow S_{i}\circ \frac{H^{T}\left(W_{i}\circ A_{i}\right)H}{H^{T}\left(W_{i}\circ \left(HS_{i}H^{T}\right)\right)H},$$

where ∘ and ÷ denote entry-wise multiplication and division for matrices, respectively.

Similarly, when we update matrix *H*,

$$\frac{\partial L(H)}{\partial H}=-4\sum_{i=1}^{t}\lambda_{i}\left(W_{i}\circ \left(A_{i}-HS_{i}H^{T}\right)\right)HS_{i}+2\eta H-B_{0},$$

where *B*_0_ ≥ 0 is the Lagrange multiplier for *H*. Thus, *H* satisfies the following equations:

$${\left(\sum_{i=1}^{t}\lambda_{i}\left(W_{i}\circ \left(HS_{i}H^{T}\right)\right)HS_{i}+0.5\eta H-\sum_{i=1}^{t}\lambda_{i}\left(W_{i}\circ A_{i}\right)HS_{i}\right)}_{jk}∙{\left(H\right)}_{jk}=0;$$

Then, we obtain the following update formulas for *H*:

$$H\leftarrow H\circ \frac{\sum_{i=1}^{t}\lambda_{i}\left(W_{i}\circ A_{i}\right)HS_{i}}{\sum_{i=1}^{t}\lambda_{i}\left(W_{i}\circ \left(HS_{i}H^{T}\right)\right)HS_{i}+0.5\eta H}.$$

#### Implementation details

SUMO is specifically designed to integrate multi-omic data for molecular subtyping. It consists of four subroutines. It allows the user to construct the multiplex network from normalized feature matrices (*sumo prepare*), tri-factorize the multiplex network to assign samples to the desired number of clusters (*sumo run*), compare the assignments to another classification using multiple metrics (*sumo evaluate*), and detect the importance of each feature towards each cluster (*sumo interpret*).

SUMO is available in the form of a command-line tool on GitHub (https://github.com/ratan-lab/sumo) and at The Python Package Index (https://pypi.org/project/python-sumo).

#### Support for missing data

Biomedical studies measure a large number of molecular parameters. Almost every dataset has missing entries. Most methods for molecular subtyping require complete data. This implies that both samples and features that have missing entries have to be removed or the missing entries have to be imputed in the preprocessing stage. SUMO takes a different approach. It scales the calculated distance between a pair of samples by the number of common features available for both samples. If sufficient overlap (by default at least 10% of features) is not found, the distance is set to *NA* (not available). A missing value in an adjacent matrix *A*_*i*_ is equivalent to a missing edge between two nodes in the multiplex network and is masked during factorization using *W*_*i*_.

#### Sparsity parameter selection

The hyperparameter *η* in cost function (Equation 1) is used to enforce the sparsity on *H* matrix. By default, we set *η* to 0.1 based on the performance on simulated datasets. This value can be optimized for the given dataset to further improve the stability of results. SUMO provides an option to run the factorization with different sparsity values and automatically select *η*, by assessing the within clusters similarities:

$$s_{\eta }=\sum_{j}\frac{\sum_{i}sim\left(C_{i},A_{j}\right)}{n_{j}^{2}}$$

where *sim*(*C_i_,A_j_*) denotes the sum of similarities for all the sample pairs in the identified cluster *C*_*i*_ given the similarity matrix *A_j_* and *n_j_* is the number of samples for the *j*^th^ data type. We then choose *η* which results in the highest *s_η_*.

#### Consensus clustering

Our solution using multiplicative rules can be sensitive to the initial conditions and the input data. Both initialization and convergence speeds are important factors to consider when formulating the appropriate factorization algorithms (Boutsidis and Gallopoulos, 2008). Our method utilizes an SVD-based initialization approach to set the initial *H* according to the average similarity matrix across all data types. This method reduces residual error and provides faster convergence than using random initialization. However, we still have to set *S_i_* randomly; as such, the algorithm does not guarantee convergence to a local minimum. Here, we set the diagonal entries of each *S_i_* to be absolute singular values, that are derived from the SVD decomposition of the corresponding *A*_*i*_ matrix. We repeat the factorization *n* times to avoid overfitting, each time including 95% of the total samples in calculating the cluster assignments from *H* and a residual error (*RE*) for that run. We create a consensus matrix *C* from these *n* assignments that is weighted to incorporate the *RE* of each factorization in a dataset with *t* data type as follows:

$$C=\frac{\sum_{x=1}^{n}C(x)*\mathrm{weight}(x)}{\sum_{x=1}^{n}\mathrm{weight}(x)},$$

where

$$M=\max_{i}RE(i),\;1\leq i\leq n$$

$$N=\min_{i}RE(i),\;1\leq i\leq n$$

$$\mathrm{weight}(x)=\frac{M-RE(x)}{M-N},\;x=1,2,\ldots ,n$$

$$RE(x)=\sum_{i=1}^{t}\lambda_{i}{∥W_{i}\circ \left(A_{i}-HS_{i}H^{T}\right)∥}_{F}^{2},\;x=1,2,\ldots ,n$$

We use the Normalized Cut clustering algorithm (Shi and Malik, 2000) on this consensus matrix to assign the final cluster labels.

#### Estimating the optimal number of clusters

Estimation of an optimal rank for NMF is a challenging problem. It is common to compare several solutions based on a clustering metric. We implement two popular metrics that leverage the consensus matrix to help the user in the determination of stable solutions to the factorization. The first metric is the cophenetic correlation coefficient (CCC). It measures the Pearson correlation between sample distances and its hierarchical clustering. A higher CCC value is considered better. The second metric is the proportion of ambiguously clustered pairs (PAC), which is defined as the proportion of the consensus matrix values in (0.1, 0.9) range. Based on our experiments, we recommend investigating factorization rank values for which the PAC score is less than 0.1, and the CCC value is high (typically >0.95). Increasing the number of repetitions of the solver can assist in the identification of the optimal number of clusters, but as we show in Figure S7A using the Acute Myeloid Leukemia (AML) dataset from benchmark data (Rappoport and Shamir, 2018), we can identify one of the stable solutions in a small number of repetitions. Similarly, we use the same dataset to show in Figure S7B that the trends observed in the PAC curve and the CCC curve are preserved for a wide range of values corresponding to the number of samples that are removed in each iteration [0,0.1]. In the current default setting, we run 60 repetitions of the solver. With each run, we randomly remove 5% of the samples, while making sure that each sample will be clustered at least once. We then create multiple weighted consensus matrices as described in the previous section, each using a random subset of runs (by default 50). While only one of the matrices is utilized to call sample labels, the CCC and PAC metrics are calculated for every one of them, providing a robust assessment of the stability of factorization results.

#### Identification of biomarkers

Once the subtypes are assigned, a frequent challenge is to identify a set of features that correlate with the cluster separation. These can be used as markers for the assignment of future samples and can aid in understanding the differences between the groups. To this end, we first train a gradient boosting classifier implemented in LightGBM (Ke et al., 2017). We use 80% of the features for training this model while performing hyperparameter optimization of the model using a random search with 5-fold cross-validation to avoid overfitting. When we have this model, we calculate the Shapley values of all features for each identified cluster. The features with a Shapley value greater than 1 are considered to be important in driving the separation of that cluster.

#### Clustering individual data types

Our approach decomposes similarity based on each data type into *HS_i_H*^*T*^ while adding sparsity to the cost function to improve separability, and uses *H* to assign labels to the samples. When using a single data type, the decomposition of the similarity matrix can simply be done into *HH*^*T*^ without the need for a data type specific *S_i_*. This formulation is equivalent to a Laplacian-based spectral clustering. So, for each data type, we converted the similarity matrix into the normalized Laplacian and determined the eigenvalues and the eigenvectors of the Laplacian. We then used the eigenvalues of the graph Laplacian and chose the number of clusters corresponding to the maximum drop-off. We finally used k-means on the matrix with the selected eigenvectors to determine the clusters based on the data type.

### QUANTIFICATION AND STATISTICAL ANALYSIS

All statistical analysis reported in this article were performed in R. The p-values for the log-rank test in Figures 3, 4, 5, S2A, and S2C were calculated using exact permutation tests (Rappoport and Shamir, 2019b) and p-values < 0.05 were considered to be statistically significant. The enrichment for discrete parameters was calculated using the χ^2^ test for independence and enrichment for numeric parameters was calculated using the Kruskal-Wallis test. The p-values for clinical enrichment were corrected using Bonferroni correction, and adjusted p-values < 0.05 were considered to be statistically significant in Figures 3 and S2C. We describe the details of the simulations in Figure 2 in the method details. All p-values in the text are reported along with the performed statistical test.

## Supplementary Material

1

2

## Figures and Tables

**Table:** KEY RESOURCES TABLE

REAGENT or RESOURCE	SOURCE	IDENTIFIER
Deposited data		
Level 3 DNA methylation, gene expression RNAseq, miRNA mature strand expression data, and survival data for TCGA	UCSC Xena Browser	https://xenabrowser.net/datapages/?hub=https://tcga.xenahubs.net:443
Processed gene expression RNAseq, copy-number data, and survival data for METABRIC	SMRT Web Application	https://bioinformatics.cse.unr.edu/software/SMRT/
Software and algorithms		
R v3.6.3	The R Foundation	https://www.r-project.org/
SUMO v0.2.6	This paper	Github: https://github.com/ratan-lab/sumo/releases/tag/v0.2.6 Zenodo: https://doi.org/10.5281/zenodo.5762331
iClusterPlus v1.26	Mo et al., 2013	http://www.bioconductor.org/packages/release/bioc/html/iClusterPlus.html
LRAcluster v1.0	Wu et al., 2015	http://bioinfo.au.tsinghua.edu.cn/member/jgu/lracluster
MCCA from PMA package v1.2.1	Witten and Tibshirani, 2009	https://cran.r-project.org/web/packages/PMA
NEMO v0.1	Rappoport and Shamir, 2019b	https://github.com/Shamir-Lab/NEMO
PINSPlus v2.0.5	Nguyen et al., 2017	https://cran.r-project.org/web/packages/PINSPlus
SNF v2.3	Wang et al., 2014	https://cran.r-project.org/src/contrib/Archive/SNFtool
CIMLR v1.0	Ramazzotti et al., 2018	https://github.com/danro9685/CIMLR
Other		
SUMO package documentation	Github	https://python-sumo.readthedocs.io
Code to reproduce comparison of various methods	This paper	Github: https://github.com/ratan-lab/sumo_analysis Zenodo: https://doi.org/10.5281/zenodo.5762339

## MOTIVATION

High-throughput experimental methods generate big high-dimensional (multi-omics) datasets. Subtyping samples/individuals on multiple data types has the potential to reveal systems-level insights and thus facilitates personal treatment of cancers and other genetic diseases. To overcome limitations in handling missing data and technical noise appearing in such datasets, we present a robust approach for integrative subtyping of multi-omics data that can extract biologically meaningful subtypes and handle noisy inputs and missing data without imputation.

## Highlights

- SUMO performs joint factorization of patient-similarity networks to determine subtypes
- SUMO can efficiently handle missing or incomplete data and technical noise
- SUMO uses consensus clustering to assign labels and can handle various data types
- SUMO identifies a subtype of LGG with a differential prognosis and GBM-like features
