# Supplementary material for: Detecting molecular subtypes from multi-omics datasets using SUMO
Source: Cell Rep Methods. 2022 Jan 14;2(1):100152. doi: 10.1016/j.crmeth.2021.100152 (PMC8865426; doi:10.1016/j.crmeth.2021.100152)
Supplement: Document S1. Figures S1–S7 [file mmc1.pdf]

Cell Reports Methods, Volume *2*

Supplemental information

Detecting molecular subtypes from multi-omics datasets using SUMO

Karolina Sienkiewicz, Jinyu Chen, Ajay Chatrath, John T. Lawson, Nathan C. Sheffield, Louxin Zhang, and Aakrosh Ratan

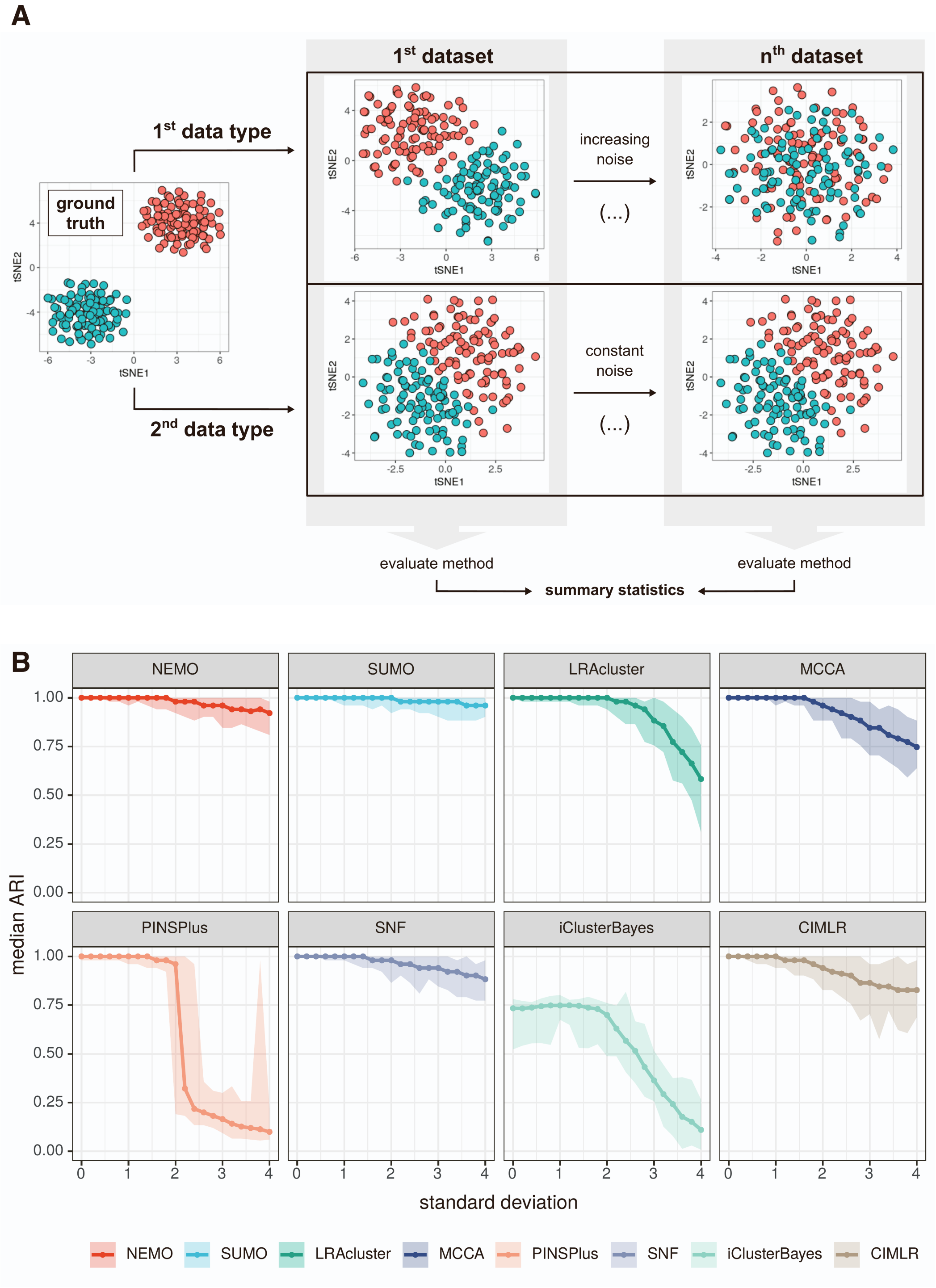

Figure S1: **Comparison of the methods on noisy data. Related to Figure 2 and simulated noisy dataset section in STAR Methods.** (A) Experimental setup to compare the various methods on noisy data. During simulation, noise in one of the data types remains constant (generated from Gaussian distribution $\mathcal{N}(\mu = 0, \sigma = 1.5)$). Another data type contains data with increasing noise, randomly generated from the Gaussian distribution as a function of standard deviation, and constant mean. Here we show two sampling points for following sets of parameters $(\mu, \sigma) \in \{(0, 1), (0, 4)\}$. (B) Adjusted Rand Index (ARI) from running the various methods on simulated noisy datasets. Datasets were created by adding either $\mathcal{N}(\mu = 0, \sigma = 1.5)$ noise (constant layer) or $\mathcal{N}(\mu = 0)$ with standard deviation $\sigma \in (0, 4)$ (layer with varying amount of noise). We report median ARI of the classification at each data point for 100 repetitions. Error ribbons display minimum and maximum ARI values.

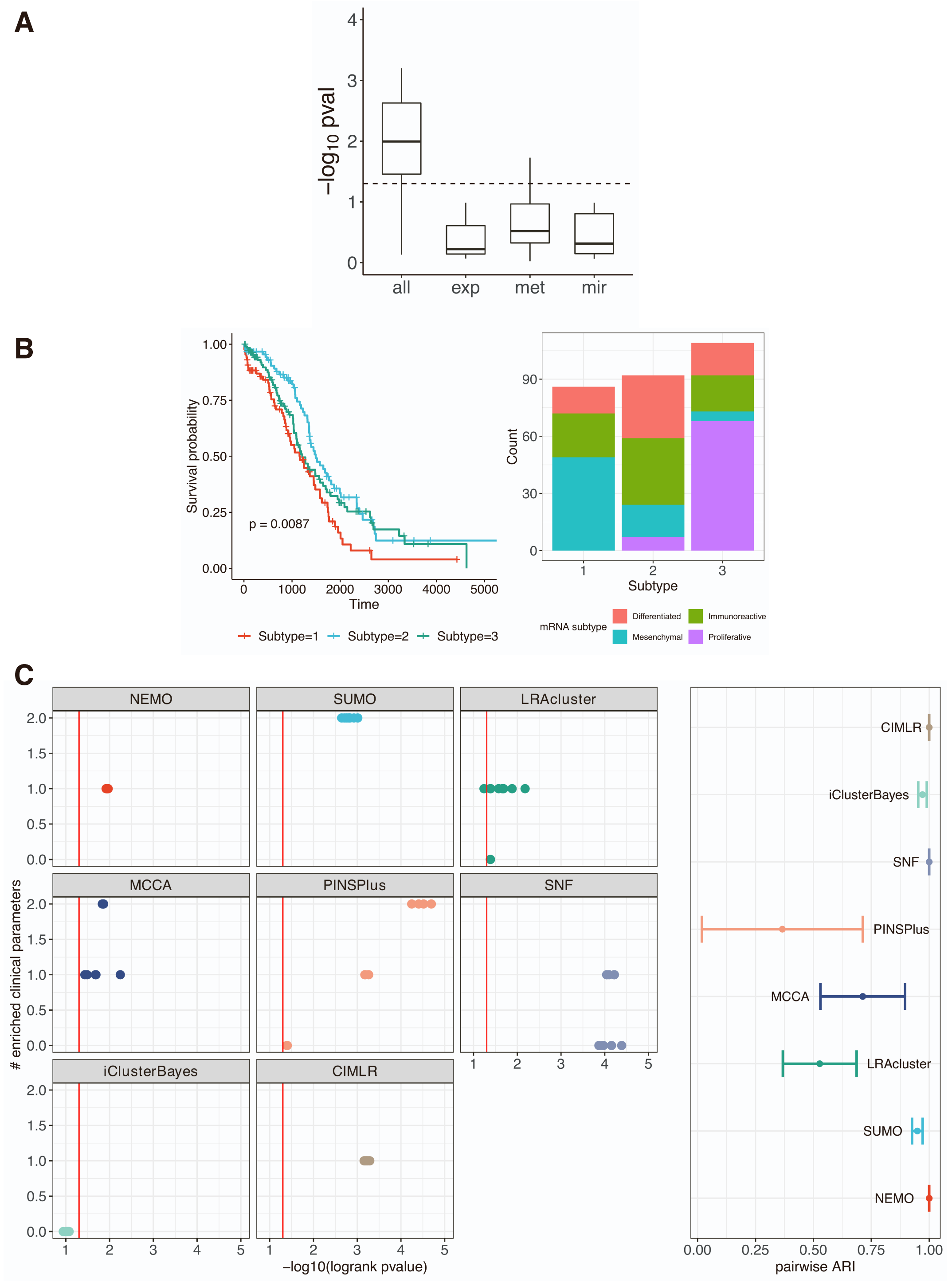

Figure S2: **Evaluation of SUMO on a benchmark. Related to Figure 3 and benchmark section in STAR Methods.** (A) We compare the p-values of the log-rank test for each data type (Exp: Gene Expression, Met: DNA Methylation, Mir: Micro-RNA) to the p-values when all the data types are integrated to show that integration of data types improves Cox p-values. (B) SUMO identifies a subgroup of patients with significant differential survival in the TCGA-OV dataset. We show the KM analysis of the identified clusters and the distribution of the patients based on the subtypes identified by using the mRNA dataset. (C) We ran each method was run 10 times on this dataset using different random seeds as input. The vertical line indicates a p-value equal to 0.05 and the right panel shows the stability of the benchmark results. We report the median and standard deviation of ARI resulting from a pairwise comparison of sample labels produced in each run of the tool.

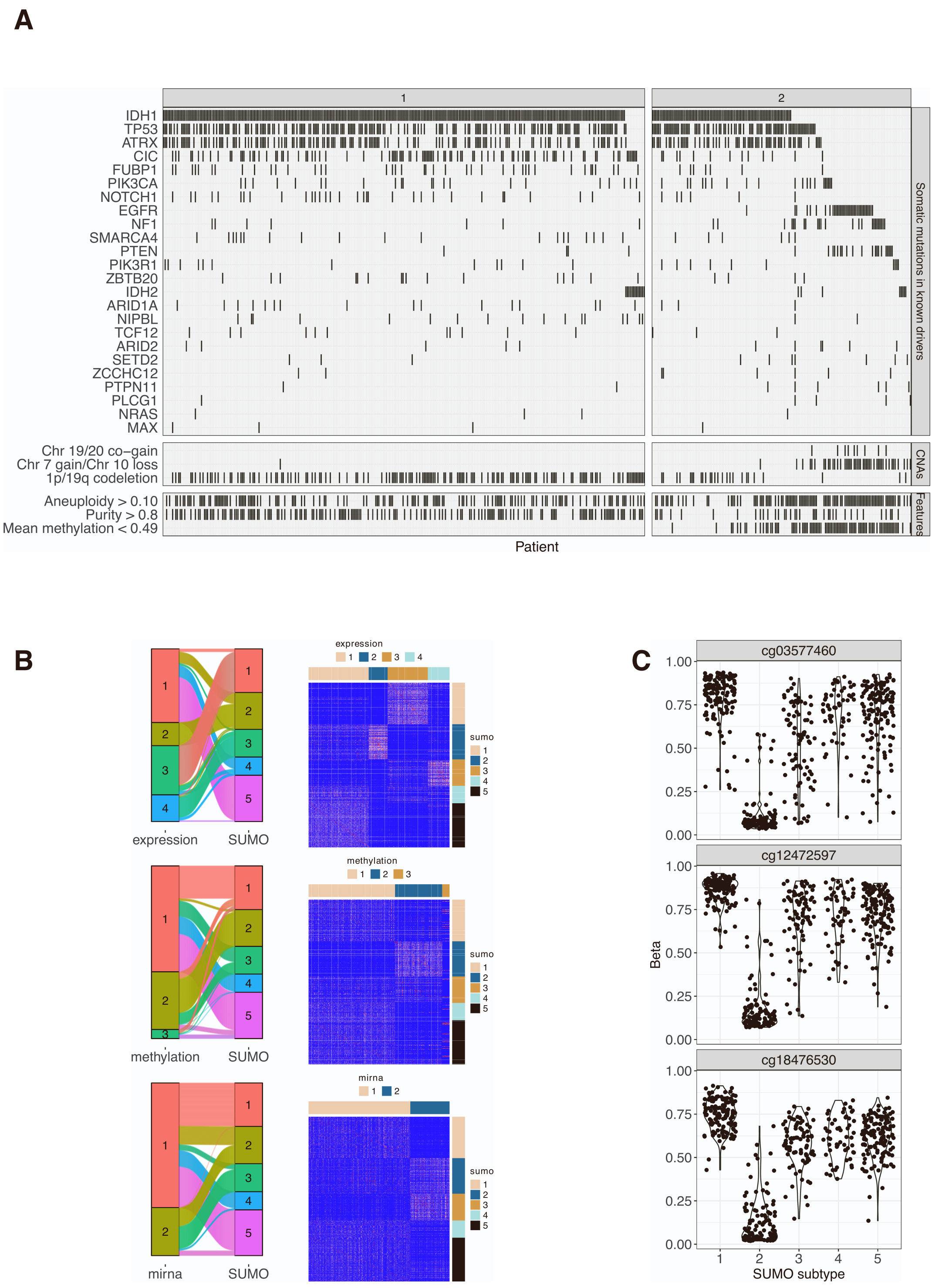

Figure S3: **SUMO applied to the LGG dataset. Related to Figure 5.** (A) Association of the 2 subtypes identified by SUMO with mutations, copy-number aberrations, and molecular features of LGGs. (B) Comparison of clustering based on individual data types to clustering after integration. We use spectral clustering to cluster each of the three data types from the TCGA-LGG dataset and compare the results to the integrative clustering done by SUMO. The order of the columns represents the groups as determined from individual data types, and the order of samples in the rows is as determined by SUMO. (C) The top three features with the potential to be biomarkers for Subtype 2. We show the violin plot of the beta values for the probes with the highest predictive values, as identified by running the interpretation module in SUMO.

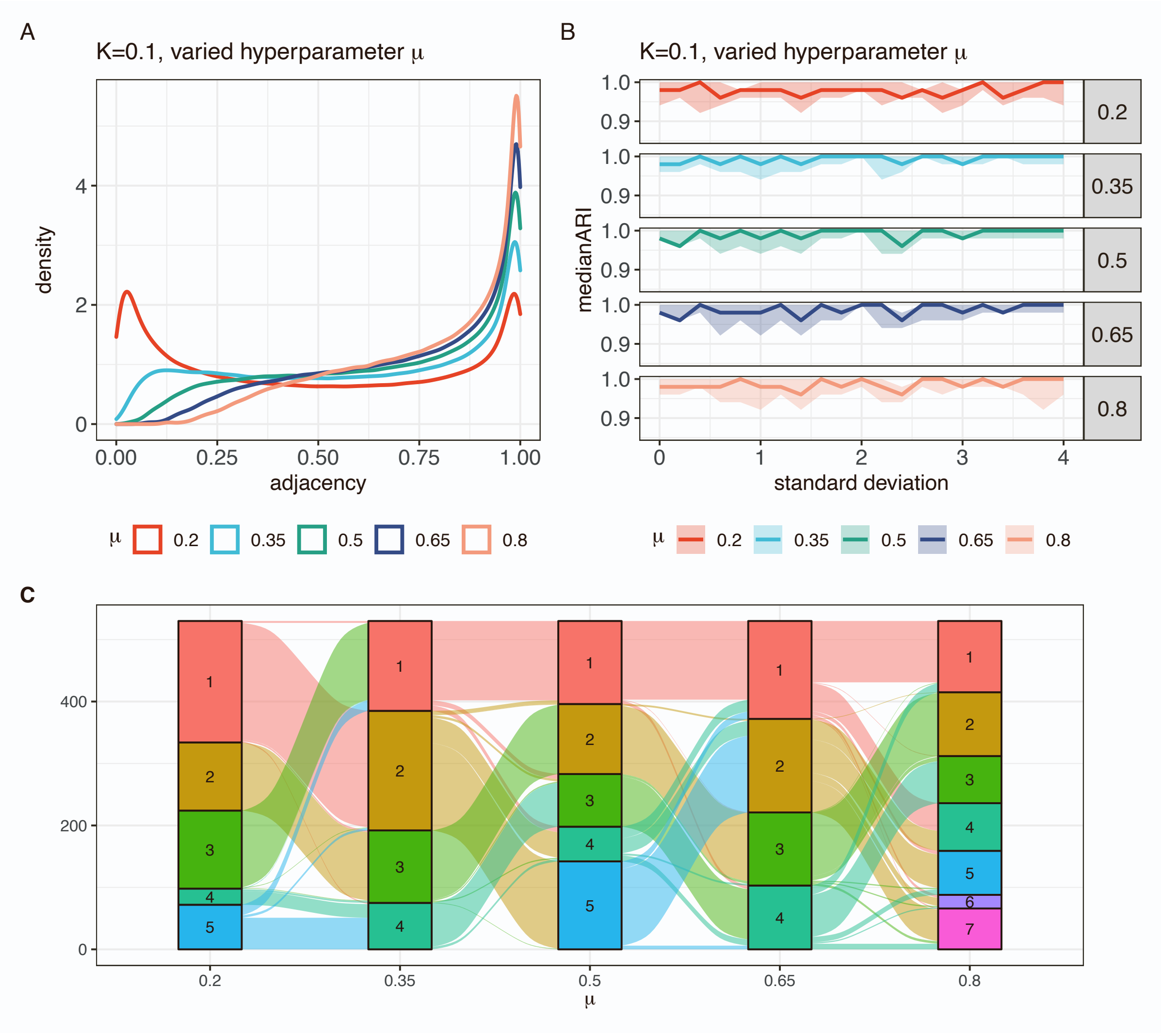

Figure S4: **Selection of μ may impact the classification results. Related to Figure 2 and the construction of similarity networks and matrices section in STAR Methods.** Here, we vary μ (the hyperparameter of the Gaussian kernel), while keeping K (proportion of nearest neighbors used for sample-sample distance normalization) constant. (A) At lower values of μ for a simple one-feature dataset, the number of pairs with lower values of similarity increases. (B) shows the comparison of final SUMO labels to ground truth for simulated datasets with varying amounts of noise. We show the median, minimum, and maximum Adjusted Rand Index (ARI) at each point for 5 repetitions. (C) shows the Sankey plot of sample membership for the optimal number of clusters at the different values of μ.

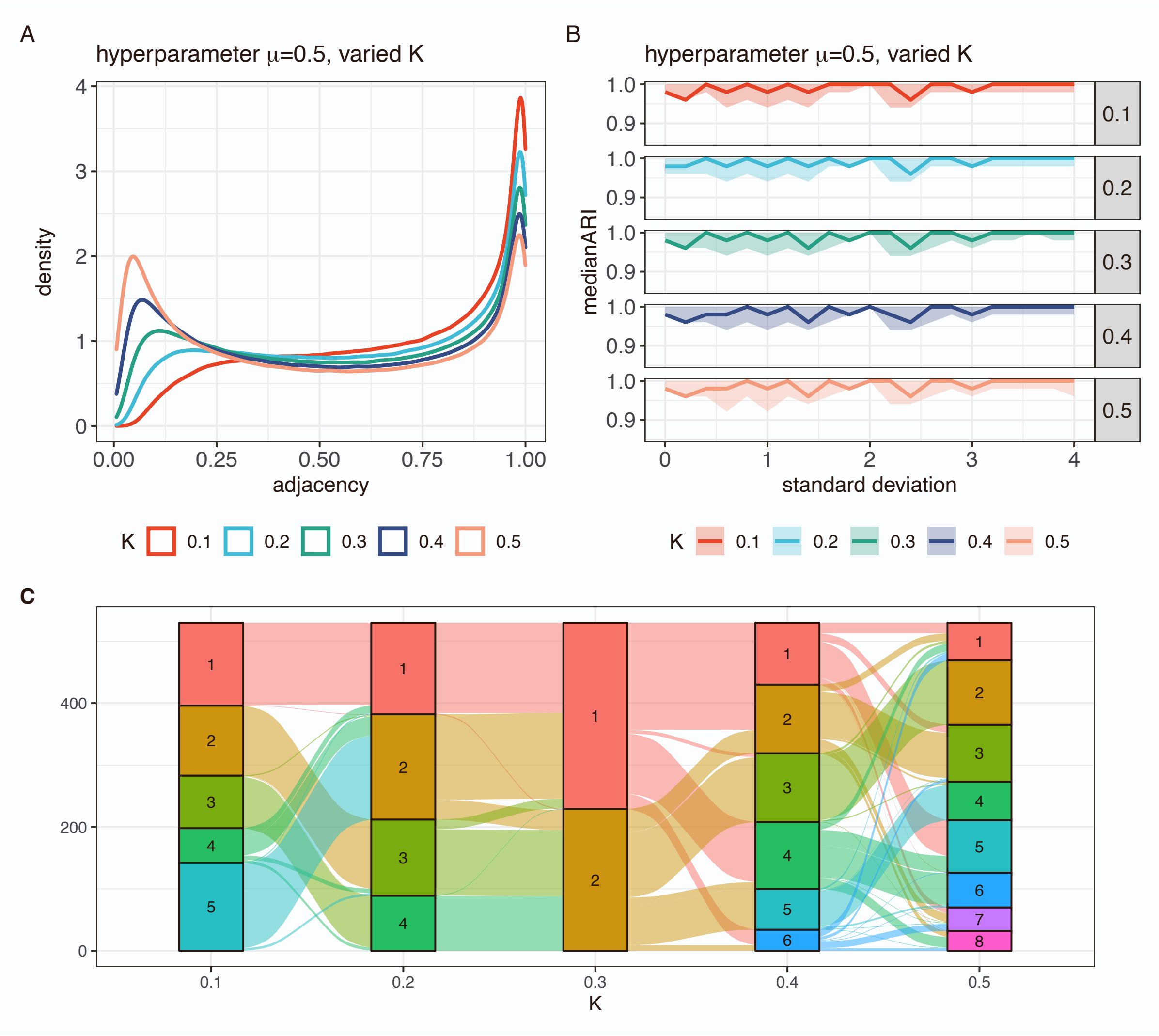

Figure S5: **Selection of K may impact the classification results. Related to Figure 2 and the construction of similarity networks and matrices section in STAR Methods.** Here, we vary K (proportion of nearest neighbors used for sample-sample distance normalization), while keeping the default value of μ. (A) At higher values of K for a simple one-feature dataset, the number of pairs with lower values of similarity increases. (B) shows the comparison of final SUMO labels to ground truth labels for simulated datasets with varying amounts of noise (see Figure 2 for dataset generation details). We show the median, minimum, and maximum Adjusted Rand Index (ARI) at each point for 5 repetitions. (C) shows a Sankey plot of sample membership for the optimal number of clusters as K is varied.

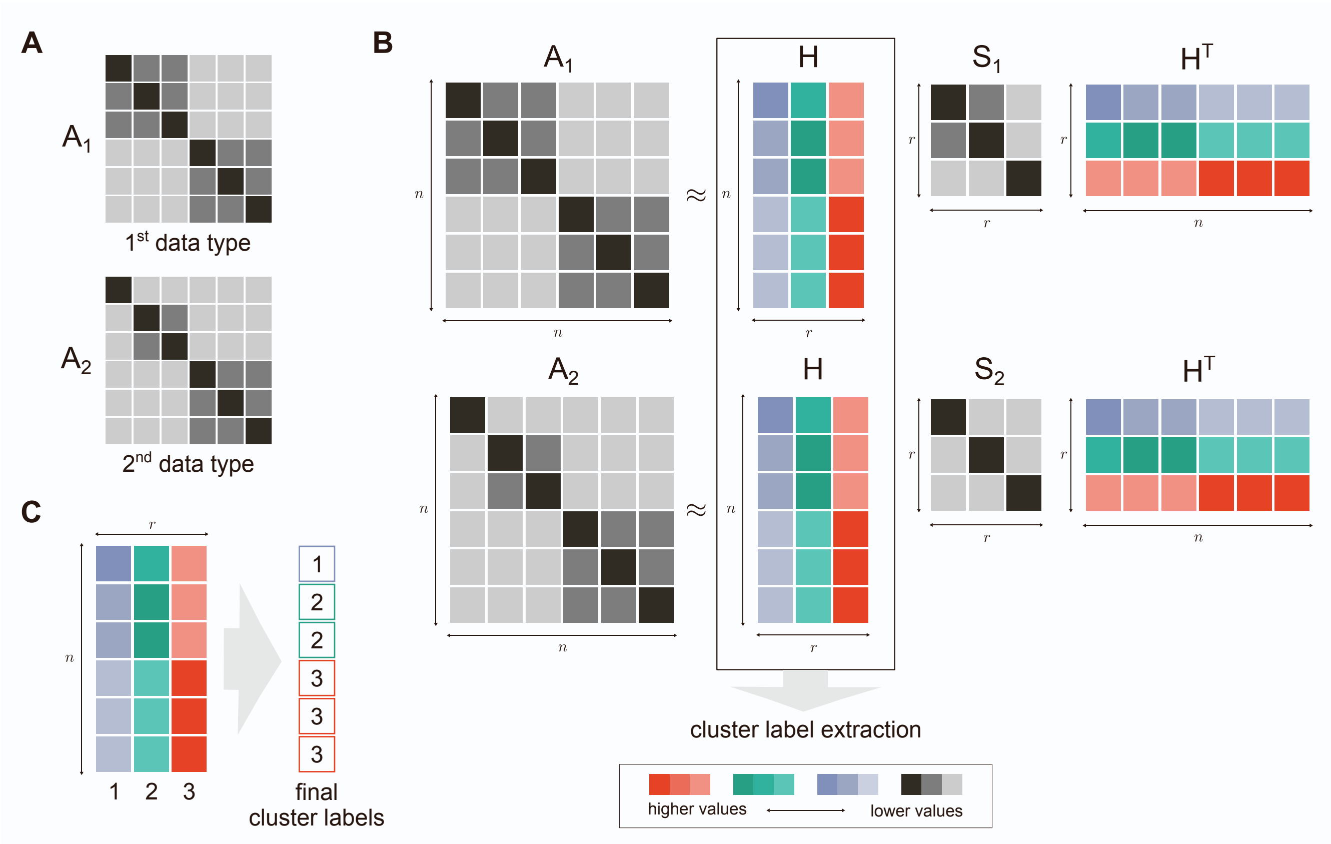

Figure S6: **Illustrative description of factorization. Related to Figure 1 and method overview section in STAR Methods.** (A) Two similarity matrices A_1 and A_2 display complementary sample-sample similarity in both data types. (B) Each similarity matrix is tri-factorized in such a way that the H matrix is shared across the data types and afterward used for cluster label extraction. Data type specific S_i matrices display relationships between clusters. (C) Final cluster labels for samples are extracted by inspecting columns containing row-wise maximum values of H matrix.

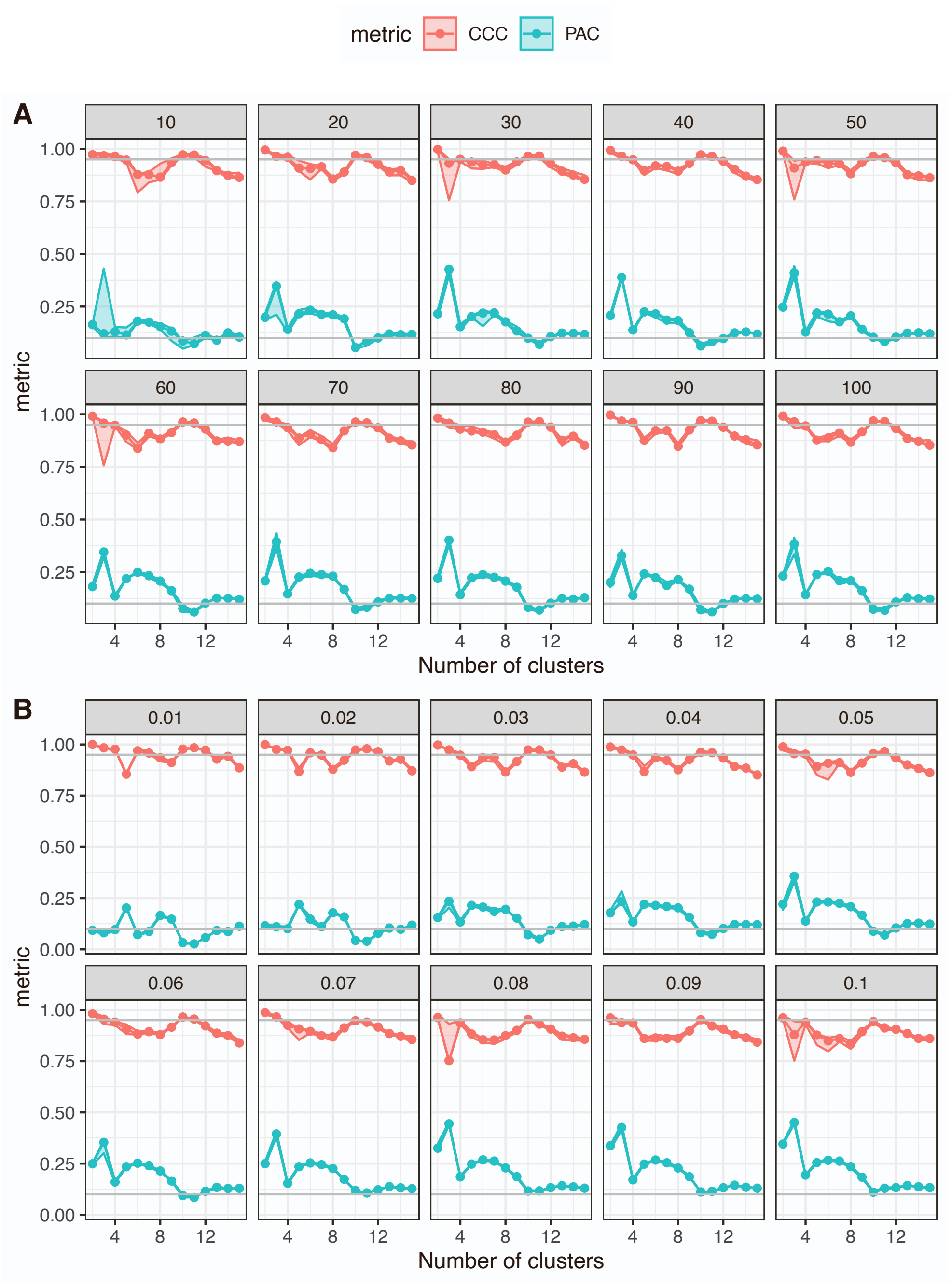

Figure S7: **SUMO is stable for a wide range of repetitions and subsets of data. Related to estimating the optimal number of clusters section in STAR Methods.** (A) Here, each facet shows the proportion of ambiguously clustered pairs (PAC) and the cophenetic correlation coefficient (CCC) curves (the minimum, median, and the maximum value of those metrics are shown for each "number of clusters") as the number of repetitions of the solver is increased. SUMO identifies either 10 or 11 as the optimal number of clusters when a small number of repetitions are run. As the number of repetitions increase, both 10 and 11 emerge as equally stable solutions. The horizontal lines correspond to values of 0.1 and 0.95. (B) Here, each facet shows the PAC and CCC curves (the minimum, median, and the maximum value of those metrics are shown for each "number of clusters") as the fraction of samples that are removed in each of repetitions of the solver is varied. SUMO identifies either 10 or 11 as the optimal number of clusters as the fraction is changed from 1% to 10% of the samples. The horizontal lines correspond to values of 0.1 and 0.95.
